# Association of Body Mass Index with Long-Term All-Cause Mortality in Patients Who Had Undergone a Vertebroplasty for a Vertebral Compression Fracture

**DOI:** 10.3390/jcm11216519

**Published:** 2022-11-02

**Authors:** Wen-Chien Wang, Yun-Che Wu, Yu-Hsien Lin, Yu-Tsung Lin, Kun-Hui Chen, Chien-Chou Pan, Jun-Sing Wang, Cheng-Hung Lee

**Affiliations:** 1 Department of Orthopedics, Taichung Veterans General Hospital, Taichung 40705, Taiwan; 2 Department of Post-Baccalaureate Medicine, College of Medicine, National Chung Hsing University, Taichung 40227, Taiwan; 3 Department of Computer Science and Information Engineering, Providence University, Taichung 43301, Taiwan; 4 Department of Rehabilitation Science, Jenteh Junior College of Medicine, Nursing and Management, Miaoli 35664, Taiwan; 5 Division of Endocrinology and Metabolism, Department of Internal Medicine, Taichung Veterans General Hospital, Taichung 40705, Taiwan; 6 Rong Hsing Research Center for Translational Medicine, Institute of Biomedical Science, National Chung Hsing University, Taichung 40227, Taiwan; 7 Department of Food Science and Technology, Hung Kuang University, Taichung 43304, Taiwan

**Keywords:** body mass index, mortality, vertebral fracture, vertebroplasty

## Abstract

We aimed to investigate the association between preoperative body mass index (BMI) and postoperative long-term mortality in patients who underwent a vertebroplasty. We retrospectively enrolled patients with a vertebral compression fracture who underwent a vertebroplasty between May 2013 and June 2020 in a medical center in Taiwan. The survival status of the study sample was confirmed by the end of March 2021. Cox-proportional hazard models were conducted to examine the effects of being overweight/obese (≥25 kg/m^2^ vs. <25 kg/m^2^) and BMI (as a continuous variable) on all-cause mortality after adjusting for age, sex, history of smoking, diabetes, hypertension, chronic kidney disease, and osteoporosis. A total of 164 patients were analyzed (mean age 75.8 ± 9.3 years, male 25.6%, mean BMI 24.0 ± 4.1 kg/m^2^) after a median follow-up of 785 days. Compared with a BMI < 25 kg/m^2^, a BMI ≥ 25 kg/m^2^ was associated with a significantly lower risk of all-cause mortality (HR 0.297, 95% CI 0.101 to 0.878, *p* = 0.028). These findings were consistent when BMI was examined as a continuous variable (HR 0.874, 95% CI 0.773 to 0.988, *p* = 0.031). A low BMI (<22 kg/m^2^) should be considered as a risk factor for postoperative long-term mortality in this ageing population.

## 1. Introduction

The global age-standardized body mass index (BMI) has continuously increased over the past decades [1]. Despite variations in the prevalence of overweight/obese people in different regions in the world, a BMI ≥ 25 kg/m^2^ has been associated with an increase in all-cause mortality [2,3]. This association may be partly attributed to the high risks of some non-communicable diseases (e.g., diabetes and cardiovascular diseases) for people who are overweight/obese. Alternatively, some studies have reported a J- or U-shaped relationship between BMI and mortality [4,5]. These findings raise the concern that a low BMI may indicate a risk of mortality, particularly in the elderly [6,7].

Vertebroplasty is commonly performed on patients with osteoporotic vertebral fractures [8,9]. Most patients who undergo a vertebroplasty are from an ageing population [8,9,10]. Although some postoperative outcomes have been investigated in patients who underwent a vertebroplasty [11,12], the risk factors associated with long-term mortality in this population are not yet clear. In a large population-based cohort study [13], a higher risk of musculoskeletal disease mortality was observed in people with a BMI < 24 (95% CI 24 to 25) kg/m^2^, the cut-off point considered the upper limit of normal [14]. Factors associated with short-term (30-day) mortality after vertebroplasty in ageing people have been investigated in several studies [15,16]. Nevertheless, the effect of BMI on the risk of long-term mortality in this population remains unknown. In this study, we aimed to investigate the association of BMI with postoperative long-term mortality in patients who had undergone a vertebroplasty.

## 2. Materials and Methods

We retrospectively enrolled patients with a vertebral compression fracture of the thoracic or lumbar spine who underwent a vertebroplasty in the Department of Orthopedics at our hospital between May 2013 and June 2020. Patients diagnosed with pathologic fractures and those with no information on the assessment of bone mineral density were excluded. This study was conducted in accordance with the Declaration of Helsinki. The study protocol was approved (approval number CE22167A) by the Institutional Review Board of Taichung Veterans General Hospital, Taichung, Taiwan.

Relevant information, including preoperative BMI, history of smoking, diabetes, hypertension, chronic kidney disease, osteoporosis, and level of vertebral fracture, was recorded from the electronic medical records. The survival status of the study population was confirmed by the end of March 2021 according to data obtained from the Ministry of Health and Welfare, ROC. Thereafter, de-identified data were used for analyses. We divided the study sample into two groups according to their BMI (≥25 kg/m^2^ vs. <25 kg/m^2^) in order to examine the effect of being overweight/obese (vs. normal weight) on the risk of all-cause mortality.

### Statistical Analysis

All statistical analyses were conducted using the Statistical Package for the Social Sciences (IBM SPSS version 22.0; International Business Machines Corp., Armonk, NY, USA). Between-group differences in categorical and continuous variables were examined using the chi-square test and independent t-test, respectively. Kaplan–Meier survival curves were plotted for the groups of overweight/obese (BMI ≥ 25 kg/m^2^) and normal weight (BMI < 25 kg/m^2^). Cox proportional hazard models were conducted to examine the effects of being overweight/obese (vs. normal weight) and BMI (as a continuous variable) on all-cause mortality with adjustments for age, sex, history of smoking, diabetes, hypertension, chronic kidney disease, and osteoporosis. The assumption of a Cox proportional hazard model was tested with scaled Schoenfeld residuals, which confirmed no violation of the assumption. The cubic spline of baseline BMI versus risk of all-cause mortality by a Cox proportional hazards model was performed as a sensitivity test. To validate our findings in the study sample, we examined the associations of preoperative BMI with all-cause mortality in another cohort of patients (validation cohort). Similar to the study sample, these patients underwent a vertebroplasty for a vertebral compression fracture of the thoracic or lumbar spine during the same period. Nevertheless, we did not have information on comorbidities (diabetes, hypertension, chronic kidney disease, and osteoporosis) in this cohort. Statistical significance was determined with a two-sided *p* value of less than 0.05.

## 3. Results

A total of 164 patients were analyzed (mean age 75.8 ± 9.3 years, male 25.6%, mean BMI 24.0 ± 4.1 kg/m^2^), and the median follow-up duration was 785 days. Table 1 shows the baseline characteristics of the study population according to their BMI. Patients who were overweight/obese (BMI ≥ 25 kg/m^2^, mean 28.2 ± 2.8 kg/m^2^) were younger, more likely to have diabetes, and less likely to have osteoporosis, compared with those who had a normal weight (BMI < 25 kg/m^2^, mean 21.5 ± 2.5 kg/m^2^). There were no significant between-group differences in the other variables at baseline.

**Figure 1:**
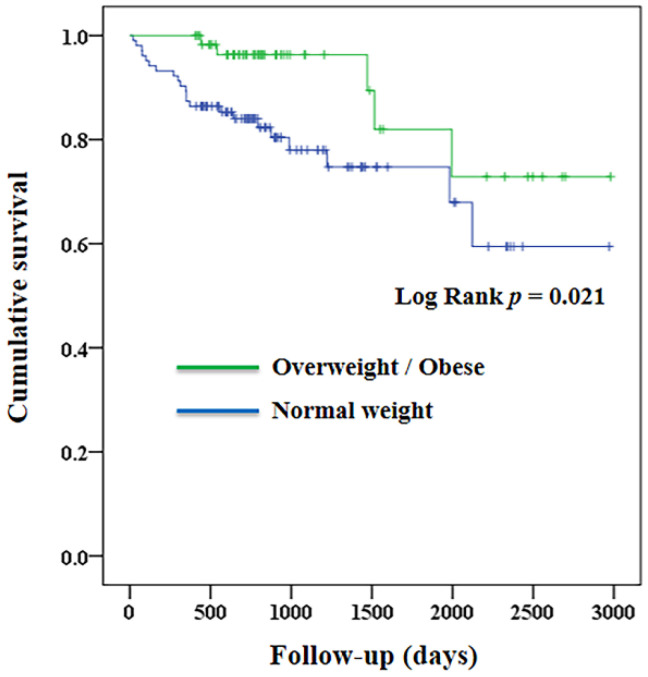
Survival curves of the study patients who were overweight/obese (body mass index ≥ 25 kg/m^2^) and normal weight (body mass index < 25 kg/m^2^).

Figure 1 shows the Kaplan–Meier survival curves in patients who were overweight/obese and those who had a normal weight. A total of 27 (16.5%) deaths were identified, and the median survival time was 785 days (interquartile range 595, 1189). We observed that the survival rate was higher in patients who were overweight/obese than it was in those with a normal weight (log rank *p* = 0.021). We examined the associations of baseline characteristics with all-cause mortality in Table 2. In univariate analysis, age, BMI (≥25 vs. <25 kg/m^2^, HR 0.336, 95% CI 0.127 to 0.890, *p* = 0.028), smoking, and chronic kidney disease were significantly associated with all-cause mortality. The associations remained significant in BMI (≥25 vs. <25 kg/m^2^, HR 0.297, 95% CI 0.101 to 0.878, *p* = 0.028), smoking, and chronic kidney disease after multivariate adjustment.

**Figure 2:**
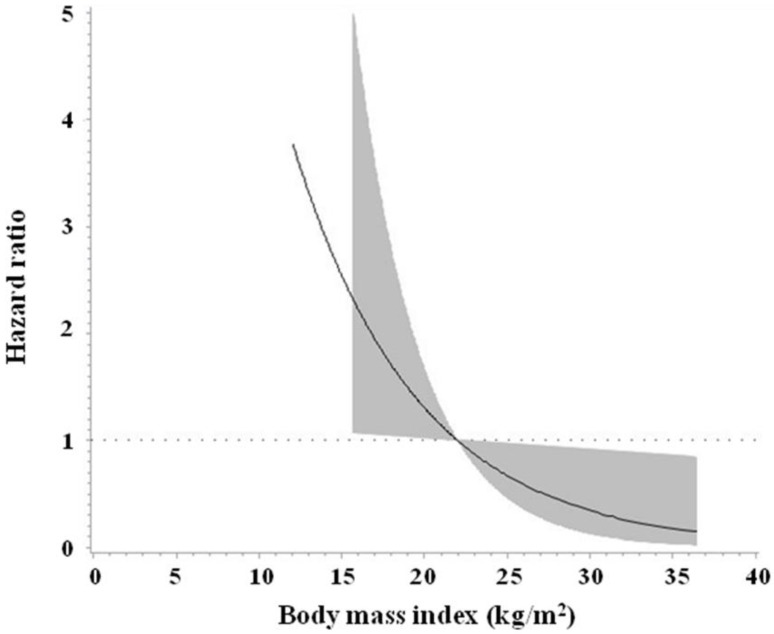
Cubic spline of body mass index versus risk of all-cause mortality in the study sample.

The findings were consistent when BMI was examined as a continuous variable. An increase in BMI was independently associated with a lower risk of all-cause mortality (adjusted HR 0.874, 95% CI 0.773 to 0.988, *p* = 0.031, Table 3). Figure 2 shows the cubic spline of BMI versus risk of all-cause mortality in the study sample. The point of BMI below which the risk of mortality started to increase was approximately 22 kg/m^2^.

We examined our findings in the validation cohort (n = 266, mean age 76.0 ± 9.4 years, male 22.9%, mean BMI 24.6 ± 4.0 kg/m^2^). A total of 101 (38.0%) deaths were identified in this cohort by the end of March 2022, and the median survival time was 1531 days (interquartile range 970, 2358). A preoperative BMI ≥ 25 kg/m^2^ (vs. <25 kg/m^2^) was associated with a lower risk of all-cause mortality (HR 0.570, 95% CI 0.377 to 0.861, *p* = 0.008, Table 4). This association remained significant after adjustment for age and sex (HR 0.588, 95% CI 0.387 to 0.891, *p* = 0.012). The findings were consistent when BMI was examined as a continuous variable (adjusted HR 0.945, 95% CI 0.895 to 0.998, *p* = 0.041).

## 4. Discussion

In this study, we demonstrated that being overweight/obese (vs. normal weight) was associated with a lower risk of all-cause mortality (HR 0.297, 95% CI 0.101 to 0.878, *p* = 0.028) in patients with a compression fracture of the thoracic or lumbar spine who had undergone a vertebroplasty during a median follow-up period of more than 2 years (785 days). Our findings were consistent when BMI was examined as a continuous variable (HR 0.874, 95% CI 0.773 to 0.988, *p* = 0.031). The point of BMI below which the risk of mortality began to increase was approximately 22 kg/m^2^ in this ageing population (mean age 75.8 ± 9.3 years). Our findings suggest that low-BMI (probably <22 kg/m^2^) ageing people who had undergone a vertebroplasty for a vertebral compression fracture may be at a higher risk for all-cause mortality.

The “obesity paradox” has been observed in patients with various medical conditions. A lower BMI was associated with a higher risk of mortality in patients with coronary artery disease [17,18] and chronic heart failure [19,20]. Similar findings were noted in patients with cancers [21,22]. Moreover, observation of the “obesity paradox” was more prominent in the elderly [23,24,25]. The aforementioned results are consistent in surgical patients [26,27]. In a large surgical cohort [27], the perioperative mortality rate was higher in patients with a BMI <25 kg/m^2^ compared with those who had a BMI ≥ 25 kg/m^2^. In line with previous reports and our findings, patients who were overweight/obese were associated with a lower risk of mortality after orthopedic surgery [28], particularly in the elderly [29,30]. People who underwent a vertebroplasty for vertebral fractures were commonly older than 65 years of age [8,9,10,31,32]. However, the effect of BMI on the risk of postoperative mortality in this population is not yet clear. Our findings suggest that patients who had undergone a vertebroplasty for a vertebral compression fracture with a BMI less than 25 kg/m^2^ (or 22 kg/m^2^) were at a higher risk of long-term mortality.

The mechanisms that account for the “obesity paradox” are not yet well understood. Patients who are underweight (BMI < 18.5 kg/m^2^) may be of a poor nutritional status. However, even if we had excluded the patients with a BMI <18.5 kg/m^2^ (n = 14) from the analyses, a lower risk of mortality was still noted in those with a BMI ≥ 25 kg/m^2^ (adjusted HR 0.311, 95% CI 0.102 to 0.951, *p* = 0.041 vs. BMI 18.5 ≤ 25 kg/m^2^). Some researchers have hypothesized that surgical patients who are overweight/obese may have a better preoperative nutritional status than those who are at a normal weight [33,34]. Additionally, there is likely to be an increase in both physiological stress and metabolic demands after surgical intervention, causing those patients with a low BMI to possibly be unable to appropriately adapt to these conditions [35], thus resulting in unfavorable outcomes. This scenario is more likely to be observed in an ageing population [36]. A low BMI in the elderly has been associated with malnutrition and sarcopenia [37,38], both of which have been associated with poor outcomes and mortality in older adults with fractures [39,40]. These findings may help explain the inverse relationship between BMI and mortality in our patients. Given that the effect of BMI on postoperative long-term mortality in patients who have undergone a vertebroplasty for a vertebral fracture has not yet been made clear, our results raise the concern that a low BMI results in adverse patient outcomes in this ageing surgical population.

Our study does have some limitations. First, this was a retrospective study with a relatively small number of study patients. The causal relationship between a low BMI and mortality risk could not be confirmed. Second, we did not have any information on the control status and medication use of some chronic diseases (e.g., blood pressure and diabetes control). Third, we did not investigate the cause-specific mortality in this relatively small study population. These factors should all be taken into account when interpreting our results. Despite these limitations, our findings underline the importance of preoperative BMI on postoperative outcomes in elderly surgical patients. This issue certainly deserves further investigations.

## 5. Conclusions

In summary, an increase in BMI was associated with a lower risk of all-cause mortality in patients who had undergone a vertebroplasty for a vertebral compression fracture. As these patients are usually older than 65 years of age, a low BMI (e.g., <22 kg/m^2^) should be considered a risk factor for postoperative long-term mortality.

## Figures and Tables

**Table 1:** Baseline characteristics of the study sample according to body mass index.

Variables	<25 kg/m^2^	≥25 kg/m^2^	*p* Value
N	103	61	
Age, years	77.2 ± 9.4	73.5 ± 8.6	0.013
Male sex, n (%)	30 (29.1)	12 (19.7)	0.180
Body mass index, kg/m^2^	21.5 ± 2.5	28.2 ± 2.8	<0.001
<18.5 kg/m^2^, n (%)	14 (13.6)	---	---
Smoking, n (%)	10 (9.7)	2 (3.3)	0.126
Diabetes, n (%)	15 (14.6)	18 (29.5)	0.021
Hypertension, n (%)	52 (50.5)	35 (57.4)	0.393
Chronic kidney disease, n (%)	36 (35.0)	20 (32.8)	0.778
Osteoporosis, n (%)	83 (80.6)	38 (62.3)	0.010
Medication for osteoporosis, n (%) ^a^	68 (66.0)	36 (59.0)	0.368
Level of vertebral fracture, n (%)			0.847
T-spine	44 (42.7)	27 (44.3)	
L-spine	59 (57.3)	34 (55.7)	

Values are mean ± SD or n (%). ^a^ Bisphosphonate, receptor activator of nuclear factor kappa-B inhibitor, or parathyroid hormone.

**Table 2:** Associations of baseline characteristics with all-cause mortality of the study sample.

	Univariate Analysis		Multivariate Analysis ^a^	
	HR (95% CI)	*p*	HR (95% CI)	*p*
Age, year	1.052 (1.003, 1.104)	0.037	1.028 (0.979, 1.079)	0.265
Sex (male vs. female)	1.018 (0.430, 2.409)	0.968	0.297 (0.088, 1.008)	0.052
BMI (≥25 vs. <25 kg/m^2^)	0.336 (0.127, 0.890)	0.028	0.297 (0.101, 0.878)	0.028
Smoking (yes vs. no)	2.844 (1.075, 7.524)	0.035	9.012 (2.166, 37.495)	0.003
Diabetes (yes vs. no)	1.453 (0.614, 3.439)	0.396	1.518 (0.578, 3.986)	0.397
Hypertension (yes vs. no)	2.016 (0.881, 4.617)	0.097	2.216 (0.903, 5.441)	0.082
CKD (yes vs. no)	2.601 (1.211, 5.587)	0.014	3.137 (1.375, 7.157)	0.007
Osteoporosis (yes vs. no)	2.368 (0.815, 6.879)	0.113	1.326 (0.420, 4.188)	0.631

BMI, body mass index. CKD, chronic kidney disease. ^a^ Adjusted for age, sex, BMI, smoking, diabetes, hypertension, CKD, and osteoporosis.

**Table 3:** Effect of body mass index (as a continuous variable) on all-cause mortality.

	Hazard Ratio (95% CI)	*p*
Body mass index (kg/m^2^)		
Model 1	0.889 (0.808, 0.977)	0.015
Model 2	0.896 (0.810, 0.990)	0.031
Model 3	0.874 (0.773, 0.988)	0.031

Model 1, unadjusted. Model 2, adjusted for age and sex. Model 3, adjusted for variables in Model 2 plus smoking, diabetes, hypertension, chronic kidney disease, and osteoporosis.

**Table 4:** Effect of body mass index on all-cause mortality in the validation cohort.

	Hazard Ratio (95% CI)	*p*
Body mass index ≥25 vs. <25 kg/m^2^		
Model 1	0.570 (0.377, 0.861)	0.008
Model 2	0.588 (0.387, 0.891)	0.012
Body mass index (kg/m^2^)		
Model 1	0.941 (0.893, 0.992)	0.025
Model 2	0.945 (0.895, 0.998)	0.041

Model 1, unadjusted. Model 2, adjusted for age and sex.

## Data Availability

The data presented in this study are available on request from the corresponding author. The data are not publicly available due to privacy/ethical restrictions.
